# Applications of artificial intelligence in systemic lupus erythematosus: integrating multi-omics data for precision medicine

**DOI:** 10.3389/fimmu.2026.1804598

**Published:** 2026-05-07

**Authors:** Biswajit Biswas, Sana Munquad, Kamalika Roy Choudhury, Bobby J. Cherayil, Amitabha Chaudhuri

**Affiliations:** 1 Research and Development, ThinkBio.Ai, Kochi, Kerala, India; 2 Mucosal Immunology and Biology Research Center, Massachusetts General Hospital, Boston, MA, United States

**Keywords:** artificial intelligence, disease stratification, foundation models, machine learning, multi-omics, precision medicine, SLE, Lupus

## Abstract

Systemic lupus erythematosus (SLE) is a clinically and biologically heterogeneous autoimmune disease in which patients with similar or disparate clinical phenotypes can exhibit distinct molecular drivers. This heterogeneity limits the utility of traditional, largely linear, analytic approaches and contributes to variations in diagnosis, prognosis, and therapeutic outcome. Artificial intelligence (AI), including machine learning (ML) and deep learning (DL), offers a complementary framework for extracting nonlinear patterns from complex biomedical datasets and for translating high dimensional molecular measurements into actionable clinical signatures. Here, we review how supervised and unsupervised ML methods are being applied to SLE, with a focus on multi-omics integration across genomics, transcriptomics, proteomics, and metabolomics. In this review, we specifically focus on how these approaches can define molecular endotypes, explaining heterogeneity in disease course and therapeutic response. We summarize evidence that AI-driven endotyping can reproducibly separate patients into molecularly defined subgroups dominated by distinct immune signatures and facilitate biomarker discovery and improved risk modelling. We highlight the limitations of conventional clinical phenotyping and the need for AI-driven biologically grounded patient stratification. Importantly, we frame these advances within a systems biology perspective, in which AI-driven integration of multi-omics and clinical data enables a unified approach to disease diagnosis, molecular stratification, prognosis, and therapeutic response prediction in SLE. Despite these advances, most AI-derived predictors remain research-grade, and require prospective multi-center validation and explicit demonstration of clinical utility before routine deployment. If key barriers to clinical translation can be overcome, AI-based methods hold promise for achieving truly personalized approaches in the management of SLE.

## Introduction

Artificial intelligence (AI) represents a transformative computational technology that aims to create systems capable of performing tasks that typically require human intelligence. In the context of biomedical research, AI has emerged as a powerful tool to address complex diagnostic and therapeutic challenges, particularly in diseases characterized by significant heterogeneity and complexity such as systemic lupus erythematosus (SLE). Machine learning (ML), a subdomain of AI, learns task-specific patterns from data, but this learning is often narrow and does not transfer robustly across different problems or data distributions without retraining or fine-tuning (1, 2). ML models learn from three types of learning paradigms. Supervised learning trains algorithms using labeled datasets where both inputs (features) and correct outputs (labels) are known, enabling building of diagnostic models and disease activity prediction. Unsupervised learning works with unlabeled data to discover hidden patterns and patient subgroups (endotypes) without predetermined outcomes. Reinforcement learning trains algorithms through trial-and-error feedback (rewards and penalties for correct and incorrect predictions respectively) and can be applied to a wide range of biological problems to gain insights from complex data. Deep learning (DL) refers to neural network architectures with multiple layers that can operate across all three learning paradigms, automatically extracting hierarchical representations from raw data to model complex, non-linear patterns. Like ML, Foundation Models (FMs) also belong to a branch of AI. These models learn to decipher biological variability by training on large and diverse datasets and transferring the learning across multiple tasks and categories with minimal or no retraining (1, 2). Simply stated, an ML model trained to identify cats would need to be retrained to identify dogs (3), whereas an FM model will discriminate cats from dogs without retraining.

AI represents the overarching goal of intelligent behavior and ML/FM provide the methodology to achieve it through learning from data (1, 4). In **Table 1** AI/ML approaches in SLE range from supervised and unsupervised methods to DL and emerging FMs. While traditional methods enable diagnosis and stratification, transformer-based FMs, such as *Bulkformer*, *scGPT*, *scPRINT*, and *Geneformer* learn transferable representations from large-scale datasets, supporting integrative and predictive analyses across molecular layers. The table also summarizes different data models, their learning mechanisms and their application in studies of SLE. Supervised ML models have been extensively used in SLE research for diagnosis and to predict disease activity from clinical and molecular data (16). While simpler, interpretable models (logistic regression, tree-based ensemble models) facilitate biological insight and clinical trust, more complex models (deep neural network and XG-boost) often achieve higher accuracy at the cost of transparency. This balance between performance and interpretability is a recurring theme in AI-driven lupus research.

**Table 1:** AI and ML models in SLE research.

Data model	Example	Definition	Learning mechanism	SLE research application	References
Machine learning (Supervised)	SVM, RF, DT, KNN, GNB	Training on labeled datasets with known inputs and outputs	Learns task-specific patterns to classify or predict outcomes	Diagnosis, disease activity prediction (e.g., SLERPI), and flare prediction	(5, 6)
Machine Learning (Unsupervised)	PCA, t-SNE	Working with unlabeled data to find inherent structures	Discovers hidden patterns and natural groupings without predetermined outcomes	Patient endotyping (molecular subgroups) and WGCNA-based gene clustering	(7–9)
Deep Learning (DL)	Autoencoder, ANN, CNN, RNN, LSTM, GRU	Subset of ML using multi-layered neural networks. Defines the architecture of the model	Automatically learns hierarchical, non-linear representations from raw data	Image-based biopsy analysis, disease classification, drug response prediction, and complex multi-omics integration	(10, 11)
Foundation Models (FM)	Bulkformer, scGPT, scPRINT, Geneformer LLMs	Subset of DL; Foundation Models (including LLMs) represent a shift toward generalized, adaptable AI, trained on large-scale, diverse datasets	Learns broad biological representations that transfer across tasks without retraining	Harmonizing datasets across platforms and identifying conserved immune states, Cell type annotation, Drug response prediction, GRN analysis	(12–15)
ARTIFICIAL INTELLiGENCE (AI)	Encompasses all data models as shown above. Built using DL, implemented using transformer architectures, classified as FMs and capable of generalization, reasoning and learning across different tasks				

AI, Artificial Intelligence; ANN, Artificial Neural Network; CNN, Convolutional Neural Network; DL, Deep Learning; DT, Decision Tree; FM, Foundation model; GNB, Gaussian Naive Bayes; GRN, Gene Regulatory Network; GRU, Gated Recurrent Unit; KNN, K, Nearest Neighbor; LLM, Large Language Model; LSTM, Long Short Term Memory; ML, Machine Learning; PCA, Principal Component Analysis; RF, Random Forest; RNN, Recurrent Neural Network; scGPT, single, cell Generative Pretrained Transformer; scPRINT, single, cell Probabilistic Representation Integration; SLERPI, Systemic Lupus Erythematosus Risk Probability Index; SVM, Support Vector Machine; t, SNE, t, Distributed Stochastic Neighbor Embedding; WGCNA, Weighted Gene Co, expression Network Analysis.

The current state of application of ML approaches on omics data in lupus is briefly summarized (**Table 2**). Guy et al. combined small rule-based models (bootstrapping) with alternate decision trees on genomics data and identified single nucleotide polymorphisms (SNPs) associated with lupus risk (17). Similarly, using multiple ML classifiers Chung et al. showed that genomic data could improve lupus identification when combined with clinical records in anti-nuclear antibody positive patients (18). Guo et al. reported exhaustion in T_reg_ cells mediated by chromatin accessibility by applying unsupervised ML models on single-cell epigenome and transcriptome data (25). Similarly, by combining single-cell transcriptome and proteome in lupus, IFN-driven alterations across T and NK cells were reported by Trzupek et al. (41). Many studies applying unsupervised and supervised ML models have predicted lupus phenotype from transcriptomic data (27) or resolved pediatric SLE into subtypes from gene expression programs (28). Additionally, multiple studies using whole blood transcriptomic data have identified disease-activity progression groups (29), inactive from active lupus (16) and patient subsets that were not obvious from standard clinical grouping (7).

**Table 2:** AI/ML and FM applications across molecular and immunologic phenotyping layers in SLE.

Omics layer	Data type	AI/ML applications	Key findings in SLE	Strengths	Limitations	Representative references
Genomics	SNPs, GWAS, polygenic risk scores	Risk prediction, susceptibility gene prioritization, ancestry stratification	Identifies inherited risk structure and susceptibility loci; useful for baseline risk modeling	Useful for risk estimation and genomic stratification	Limited ability to explain disease heterogeneity, activity, or treatment response when used alone	(17–20)
Epigenomics	DNA methylation, histone marks, regulatory signatures	Biomarker discovery, disease classification, molecular subtyping	Identifies disease-associated regulatory signatures and subtype-linked alterations such as *EGR3* and *SYNGAP*	Captures environmentally influenced and dynamic regulatory changes	Small cohorts, cell-type variability, limited validation across independent datasets	(21–24)
Chromatin accessibility	ATAC-seq, enhancer/promoter accessibility, TF-binding landscapes	Regulatory network inference, transcription factor activity prediction, cell-state program identification	Links genetic susceptibility to downstream transcriptional regulation and immune dysregulation	Strong mechanistic interpretability	Limited disease-specific datasets, high dimensionality, largely exploratory use in SLE	(24–26)
Transcriptomics	Bulk RNA-seq, scRNA-seq, blood gene-expression profiles	Endotyping, disease activity prediction, flare prediction, treatment-response modeling	Identifies reproducible molecular endotypes and interferon-driven signatures; strongest evidence for clinically relevant heterogeneity	High predictive performance, robust datasets, strong biological interpretability	Batch effects, cohort variability, limited prospective validation	(7, 16, 27–31)
Proteomics: Autoantibody profiling	Autoantigen arrays, serologic autoantibody signatures	Patient classification, immune profiling, subgroup identification	Defines clinically relevant subsets beyond traditional markers such as anti-dsDNA and anti-Sm	Direct clinical relevance and diagnostic value	Assay variability, limited standardized large-scale datasets	(32, 33)
PROTEOMICS: SERUM/plasma proteomics	Cytokines, complement proteins, circulating mediators, serum/plasma proteomic panels	Biomarker discovery, disease monitoring, flare/activity prediction	Identifies biologically distinct subgroups and predicts disease activity, flares, and organ involvement	Strong translational potential for biomarker development	Inter-platform variability, limited longitudinal validation	(34–37)
Proteomics: Cellular proteomics or immune phenotyping	Flow cytometry, CyTOF, immune-cell protein profiles	Immune cell phenotyping, clustering, disease stratification	Defines immunophenotypic subsets linked to disease activity and flare risk; supports integrative subgroup discovery	High biological resolution and mechanistic relevance	Technical complexity, lack of standardized pipelines, small cohorts	(38–40)
Cross-modal/multi-omics integration	Combined genomic, epigenomic, transcriptomic, proteomic, metabolomic, and clinical data	Integrated diagnosis, endotyping, pathway discovery, outcome prediction	Identifies interferon-driven and oxidative stress–related subtypes and validates shared signatures across layers	Captures disease complexity better than single-omics approaches	Missing data, batch effects, lack of standardized pipelines, limited external validation	(41–44)
Foundation models (emerging)	Large-scale transcriptomic, single-cell, cytometry, imaging, and multimodal datasets	Transfer learning, multi-task prediction, cross-cohort generalization	Potential to unify diagnosis, stratification, and treatment prediction by learning shared biological representations	May improve robustness and generalizability across tasks and cohorts	Early-stage in SLE, limited prospective validation, sensitivity to batch effects, benchmarking still evolving	(12–14, 45–48)

In these studies, a wide range of ML algorithms including random forest (RF) models, support vector machines (SVM), logistic regression, and neural networks have been successfully applied to whole-blood RNA-sequencing data for SLE diagnosis, patient stratification, and prediction of treatment response (7, 16, 27, 49). Chen et al. (50) developed an ML-based flare prediction system [combined XGBoost model and the SRSPM (simplified risk score prediction model)] for lupus nephritis (LN) using dynamic clinical and serological variables, demonstrating high sensitivity and specificity in distinguishing active from quiescent disease. Complementing these supervised approaches, unsupervised methods such as hierarchical clustering and weighted gene co-expression network analysis (WGCNA) have enabled the discovery of molecular subtypes without reliance on predefined clinical labels (7, 8). William et al. identified three distinct molecular clusters characterized by interferon-, neutrophil-, and lymphocyte-driven signatures using an unsupervised method to mechanistically define distinct SLE endotypes (7). These endotypes were associated with differential enrichment of key immune-related genes, including *TNFSF13B* (*BAFF*), *TNFSF10* (*TRAIL*), *TLR7*, and *IFNAR1*, linking cluster identity to established lupus-relevant pathways. The identification of these biomarkers further supports the biological plausibility of ML-derived stratification and its relevance to disease mechanisms as well as therapeutic interventions using targeted antibodies and small molecule inhibitors (51). Integration of ML-based learning with classical approaches highlights that core disease mechanisms are context-dependent, rather than uniform across all patients. For example, interferon signature is influenced by ancestry and other covariates, supporting the idea that the disease-driven biology is influenced by population-level modifiers (52, 53).

Besides genome, epigenome and transcriptome, many studies have utilized proteomics data, including autoantigens, autoantibodies and serum plasma proteins for early lupus diagnosis, molecular phenotyping and linking circulating proteins to cell-state changes in progressive disease (34, 35, 42). Application of ML model on circulating cytokines have led to the discovery of cytokine signatures associated with SLEDAI phenotype (36).

Taken together, these findings demonstrate that genome, epigenome, transcriptome and proteome-based ML-driven stratification provides a powerful framework for understanding disease heterogeneity in SLE. By systematically linking transcriptomic structure, immune mechanisms, and clinical outcomes, ML-enabled approaches solidify our understanding of SLE pathogenesis and provide a foundation for applying precision medicine strategies for lupus.

**Figure 1:**
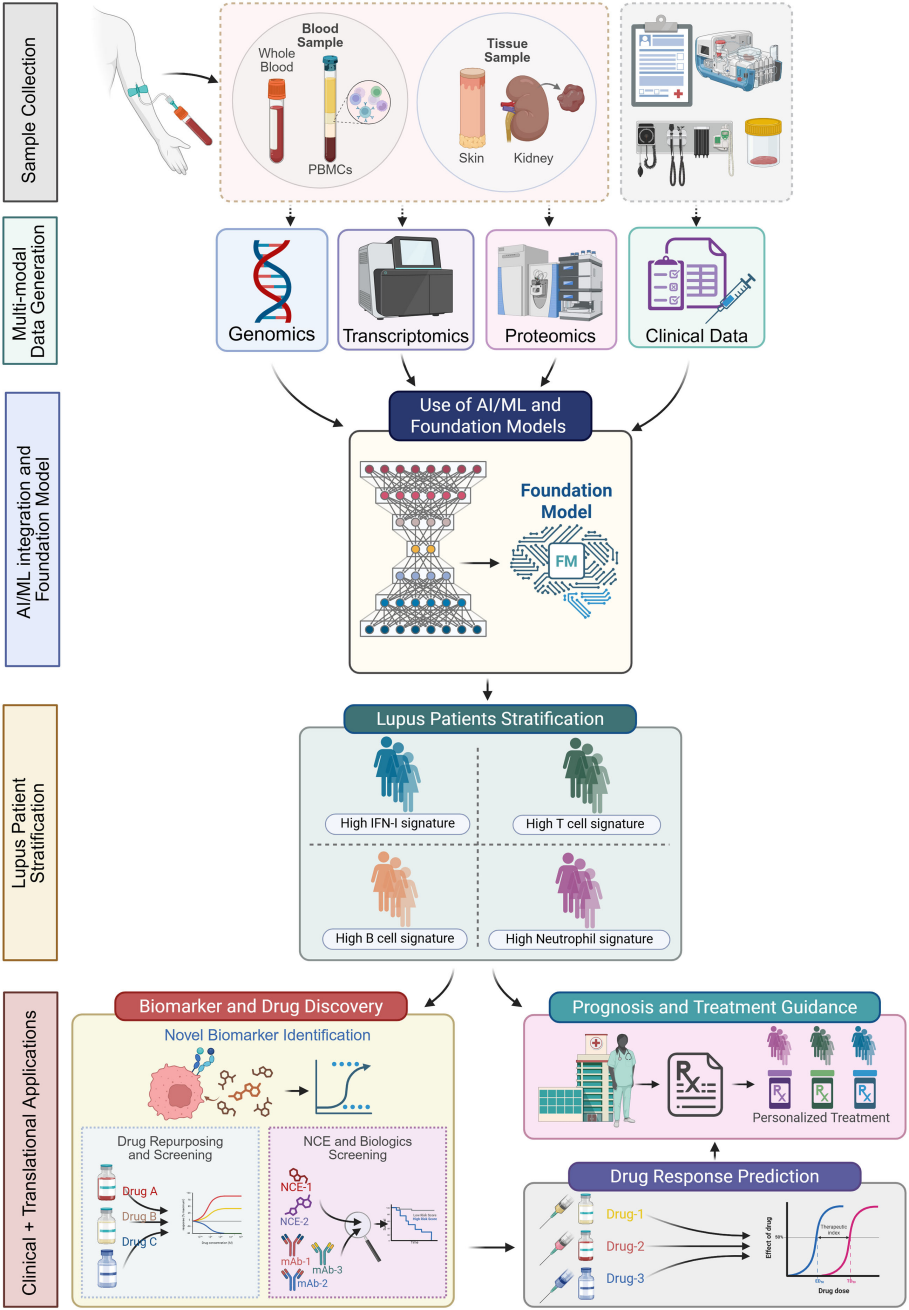
AI-driven multi-omics framework for lupus patient stratification, biomarker discovery, and precision therapeutics. The schematic illustrates an integrated workflow from sample collection to generation of data and analysis of data to arrive at clinical utility. Framework for analyzing multiomics data using ML and AI models are shown. Model outputs enable stratification of lupus patients, discovery of biomarkers, disease prognosis and treatment guidance. Finally, predictive modelling of drug response links molecular signatures to expected therapeutic efficacy, enabling rational selection and optimization of treatments for individual patients.

The power of AI is best realized when it integrates multi-omics data together with the underlying biological knowledge these datasets encode, combining complementary layers of information such as genomics (DNA variants), transcriptomics (gene expression), proteomics (protein abundance, post-translational modification), and metabolomics (small molecule metabolites) (**Figure 1**). Each data layer reveals different aspects of disease biology. ML models that integrate multiple molecular layers with thousands of measured variables are likely to introduce noise and therefore require dimensionality reduction by feature selection using Principal Component Analysis (PCA) or Distributed Stochastic Neighbor-Embedding (t-SNE). By using these preprocessing steps molecular data are combined with clinical and laboratory variables to improve discrimination compared with single-modality models (1).

SLE is well suited for AI approaches because of its marked clinical and molecular heterogeneity (54). Disease manifestations vary from mild cutaneous involvement to severe organ-threatening complications including nephritis and neuropsychiatric disease. Patients’ responses to identical treatments vary widely, and disease courses are unpredictable. Traditional statistical methods struggle with this heterogeneity since they assume homogeneous populations and linear relationships. ML and other AI approaches excels at uncovering nonlinear patterns, patient subgroups, and complex interactions (55).

AI-based approaches are aimed at identifying novel disease severity biomarkers, predicting flare risk, facilitating earlier diagnosis through pattern recognition, and discovering molecular endotypes potentially amenable to tailored therapies (5).

While recent reviews have addressed AI and high-dimensional technologies in SLE broadly (2, 56), the current article takes a more focused approach. We examine how AI-driven multi-omics integration can resolve biological heterogeneities in SLE through molecular endotyping, and how the resulting frameworks may advance diagnosis, patient stratification, prognosis, and therapeutic selection. We further highlight the use of FMs as an emerging approach for unifying transcriptomic, single-cell, imaging, and clinical data within a precision medicine framework, positioning AI not as a collection of isolated predictive tools, but as a systems-level strategy for modelling the interconnected immune dysregulation that underlies SLE pathogenesis.

## Unrealized therapeutic efficacy in SLE in the context of molecular heterogeneity

The therapeutic management of SLE has expanded considerably over the past two decades, reflecting advances in understanding disease pathophysiology and the availability of both conventional and targeted therapies. Current treatment strategies aim to induce remission or maintain low disease activity while minimizing organ damage and treatment-related toxicity. Despite this progress, clinical responses remain highly variable, underscoring the fundamental challenge posed by the molecular and immunological heterogeneity of the disease (57).

Conventional immunosuppressive therapies, including glucocorticoids (such as prednisone and methylprednisolone), antimalarials (hydroxychloroquine), and broad immunosuppressants (mycophenolate mofetil, azathioprine, and cyclophosphamide), continue to form the backbone of SLE management and are effective in controlling inflammation across diverse clinical phenotypes (58). However, their largely non-selective mechanisms, cumulative toxicities including steroid-induced osteoporosis and increased infection risk, and inconsistent durability of response highlight their limitations, particularly in patients with organ-threatening or refractory disease (59). The emergence of biologic and targeted therapies has partially addressed this gap by aligning treatment with dominant pathogenic pathways in specific patient subsets. Several targeted therapies have demonstrated meaningful clinical benefit in SLE, particularly in patients with refractory disease or lupus nephritis. B cell-depleting agents, such as the anti-CD20 monoclonal antibody rituximab, directly eliminate B cells, whereas BLyS/BAFF inhibitors, such as belimumab and tabalumab, reduce B cell survival and activation. On the other hand, type I interferon signaling can be blocked by anifrolumab, a monoclonal antibody targeting the type I interferon receptor (60). Nevertheless, even these targeted approaches fail to produce uniform responses, and many patients cycle through multiple therapies with limited or transient remission.

This variability in therapeutic response reflects long-standing gaps in the ability to define SLE at a molecular level. Historically, treatment decisions have relied primarily on clinical manifestations and a limited set of serologic biomarkers, which lack sufficient sensitivity and specificity to capture disease complexity or predict treatment response (61). Advances in genomics, transcriptomics, proteomics, and metabolomics have begun to overcome these limitations by enabling high-dimensional profiling of large lupus cohorts (**Figure 1**). Analyses of these datasets, often supported by ML approaches, have revealed distinct molecular subgroups characterized by differential activation of pathways such as type I interferon signaling, B-cell dysregulation, and myeloid-driven inflammation. FMs are extending these findings to prediction of disease progression and patient outcomes (62). The discovery of disease features across different lupus datasets are beginning to reveal a biological framework for the observed clinical heterogeneity and drug response.

Importantly, population-scale genetic studies indicate that inherited genetic variation alone explains less than 10% of SLE susceptibility, suggesting that disease heterogeneity arises from a complex interplay of molecular, environmental, and lifestyle factors (63, 64). Together, these observations illustrate why uniform therapeutic strategies, whether non-targeted or targeted, remain insufficient for many patients and highlight the need for molecularly informed, data-driven approaches to align therapies with patient-specific disease biology, an area that is rapidly advancing because of the new technologies (56).

Traditional approaches to SLE classification and management have relied primarily on clinical phenotyping, based on organ involvement, serologic markers, and disease activity indices. While useful, this framework fails to capture the underlying biological heterogeneity of the disease, as patients with similar clinical features may be driven by fundamentally distinct immunological mechanisms and exhibit markedly different responses to therapy. This limitation has led to increasing recognition that molecular phenotyping (endotyping) which is defined by gene expression patterns, immune pathway activation, and multi-omic signatures, provides a more mechanistically informative framework for understanding disease variability. In this context, ML and other AI approaches offer powerful tools to identify such molecular endotypes and link them to clinically relevant outcomes, forming the basis for precision medicine in SLE. In this review, we therefore examine not only the conceptual value of AI in SLE, but also the current state of the field across the principal molecular phenotyping domains such as genomics, epigenomics, chromatin accessibility, transcriptomics, and proteomics, with emphasis on biological insight, and translational potential.

## Utilization of AI in unveiling lupus biology

### Uncovering disease heterogeneity

ML and FM analyses of transcriptomic data have made one conclusion unequivocal: SLE is not a single molecular disease, even when patients share similar clinical features (7, 30, 65). Early transcriptomic studies demonstrated that type I interferon signaling and granulopoiesis-related gene signatures explain a substantial proportion of inter-patient variance in SLE (66). These findings provided a mechanistic foundation upon which later ML-driven studies were built, framing interferon and myeloid activation as dominant pathogenic dimensions in disease variation. However, subsequent analyses showed that these dimensions are not merely descriptive snapshots but represent relatively stable molecular states that persist across time and disease activity levels. Consistent with these early findings, ML stratification of whole-blood data consistently identifies multiple patient clusters, each characterized by distinct immune programs, supporting the concept that “one signature” is insufficient to capture the complexity of SLE (7). These observations underscore the robustness of transcriptomic heterogeneity across patients as a defining feature of the disease. The next section briefly touches upon the clinical impact of AI applications in SLE.

### Importance of patient stratification and endotyping

Building on early transcriptomic insights integrated with ML approaches, Hubbard et al. defined molecular SLE endotypes and showed that these endotypes were strongly associated with differences in clinical manifestations, disease activity, and longitudinal outcomes, thereby directly linking molecular stratification to clinically meaningful endpoints (30). Parallel integrative analyses within the same study provided additional support for the concept that clinical phenotypes, such as the occurrence of severe flares and differential responsiveness to drugs, correlated with immune signatures across independent cohorts. Thus, transcriptome-based ML stratification captures fundamental molecular structure of the disease with potential for matching patients with therapies, designing clinical trials and predicting disease progression (30). Data-driven transcriptomic analyses have transformed patient stratification in SLE by defining reproducible molecular endotypes that extend beyond heterogeneous clinical phenotypes. Unsupervised whole-blood transcriptomic studies demonstrated molecular signatures characterized by dominant immune programs involving interferons, myeloid cells/neutrophils, lymphocytes, and plasmablasts (62). Subsequent studies refined this framework by distinguishing type-1 SLE (inflammatory manifestations) and type-2 SLE (pain and fatigue) endotypes across independent cohorts (67). Collectively, these studies demonstrate that molecular endotyping provides a powerful framework for resolving SLE heterogeneity by linking immune-pathway activation to clinical variability beyond what can be achieved through phenotypic assessment alone. This mechanistic stratification, in turn, enables precision therapeutics by aligning patients with targeted treatments.

### Biomarker discovery

Disease biomarkers make clinical insights actionable by identifying patients at risk, increasing efficacy of targeted therapies and predicting disease progression. ML and DL have accelerated biomarker discovery in two ways. Firstly, the process of feature selection and compression reduces thousands of genes into smaller pathway-enriched signature panels-derived composite scores that preserve clinically relevant information. In the absence of feature selection and dimensionality reduction, biomarker discovery in lupus typically relies on gene-by-gene analyses, requiring thousands of independent statistical tests, each with modest effect sizes and high sensitivity to noise. This results in slow, unstable, and often irreproducible biomarker identification. In contrast, feature selection and compression aggregate coordinated gene activity into pathway-enriched composite scores, substantially reducing dimensionality while preserving disease-relevant biology. Hubbard et al. describe a composite scoring approach based on molecular abnormalities that supports categorization of patients by underlying biology (30). By correlating disease phenotype to enriched pathways this method facilitates accelerated biomarker discovery. Secondly, neural-network and other AI models have produced compact gene panels associated with outcomes that allow identification of predictive gene sets. For example, a neural network approach reported a 50-gene set that predicted belimumab response with cross-validated performance in a blood transcriptome context (68). It should be noted, however, that biomarker-based clinical actionability has limitations because transcriptomic biomarkers are sensitive to cohort composition, ancestry, therapy, and technical artifacts. As a result, reliability requires validation in diverse disease cohorts and identification of confounding factors that decrease the sensitivity and specificity of prediction (69). ML and DL approaches are increasingly used to identify molecular features and immune states that predict response to targeted therapies, particularly biologics.

### Treatment response prediction

Traditionally, prediction of treatment response has relied on the use of clinical features and standard serological assays. For instance, the Pooled Belimumab International SLE Study (BLISS) trial analyses established that belimumab response is enriched in serologically active SLE patients, exhibiting high-disease-activity. Patients with Safety Of Estrogens In Lupus Erythematosus National Assessment –Systemic Lupus Erythematosus Disease Activity Index (SELENA-SLEDAI) ≥10, high anti-dsDNA positivity, and low complement levels derived the greatest benefit; achieving higher SRI response rates versus placebo, with belimumab providing additive benefit alongside rather than replacing standard-of-care (70).

Predictive modelling is moving beyond clinical features toward mechanism-anchored biomarkers. Pharmacometric analyses identified baseline BLyS and anti-dsDNA as covariates influencing memory B-cell response dynamics, though their incremental predictive impact was modest (71). In lupus nephritis, baseline proteinuria emerged as the dominant stratifier of renal response probability, with belimumab exposure variation contributing minimally within the 10 mg/kg IV regimen (72). Type I interferon stimulated gene (ISG) signatures further demonstrated the ability to predict clinical response, supporting immune pathway activity scores in guiding BAFF/BLyS blockade decisions (73).

Transcriptome-driven ML approaches show particular promise by identifying baseline interferon- and B cell–related gene expression patterns associated with belimumab response, with Least Absolute Shrinkage and Selection Operator (LASSO) logistic regression applied to blood transcriptomes similarly predicting responder status (27, 68, 73, 74). Unlike conventional analyses, ML and FM-based approaches can capture clinically meaningful molecular signals in small patient subsets- a critical advantage given SLE’s marked heterogeneity. These frameworks are being extended to conventional immunosuppressants, anifrolumab, and emerging targeted therapies (75, 76).

Beyond response prediction, ML tools support upstream clinical decisions. The SLE Risk Probability Index (SLERPI), a LASSO-derived diagnostic index, achieves high accuracy in early SLE diagnosis and in identifying renal and neuropsychiatric manifestations (6). Supervised ML models integrating transcriptomic and clinical data distinguish SLE from healthy controls and related autoimmune conditions, and classify disease subtypes including lupus nephritis, neuropsychiatric SLE, and cutaneous lupus (18, 77, 78). When applied to electronic health records (EHRs), these methods enable population-level case identification, pregnancy outcome prediction, and hospitalization risk stratification (79–81).

As a note of caution, it is important to mention that currently most predictors remain research-grade, *post hoc*, retrospective, or single-cohort, and are not yet formal clinical decision tools. Prospective multicenter validation, standardized assay workflows, and demonstrated improvement in patient outcomes remain prerequisites for routine deployment (82).

### Deep learning applications

DL models have increasingly enabled a more nuanced understanding of disease heterogeneity, biomarker discovery, and patient stratification in SLE by leveraging high-dimensional multi-omics data, including transcriptomics, genomics, epigenomics, proteomics, and clinical variables. By integrating these heterogeneous data modalities, DL architectures can model complex, non-linear relationships that are difficult to capture using traditional statistical or shallow ML approaches. Consistent with this advantage, DL models have been applied to predict B-cell epitopes recognized by pathogenic autoantibodies in SLE by learning sequence and structural features associated with antibody binding. In addition, these models are applied to disease classification, response prediction, often outperforming conventional ML methods when trained using sufficient data.

Recent DL efforts in SLE have increasingly focused on interpretability and biological relevance. Rather than treating models as black boxes, these approaches aim to extract signals that map onto known immune pathways and disease processes. Studies integrating single-cell and molecular data have shown that DL can identify clinically meaningful patterns from highly complex datasets, including features associated with disease severity and outcomes, illustrating how advanced models can translate high-dimensional data into actionable insights. DL has also enabled more effective integration of diverse data types, including molecular, metabolic, imaging, and clinical information (83, 84). Multimodal approaches combining omics data with spectroscopic, histologic, or clinical inputs have demonstrated improved performance in disease classification, activity assessment, and patient endotyping, including in lupus nephritis. Importantly, these methods emphasize weighting and prioritizing biologically informative features, supporting more robust and reproducible disease stratification across heterogeneous patient populations.

Beyond molecular profiling, DL has shown promise in image-based applications relevant to routine clinical practice. In lupus nephritis, DL models applied to digitized kidney biopsy images have achieved high agreement with expert pathologists in identifying glomerular lesions, helping to reduce inter-observer variability and standardize histopathologic assessment. More recent work suggests that combining imaging with longitudinal clinical data may enable earlier detection of renal flares and more dynamic disease monitoring, extending beyond static biopsy-based evaluations (10). Li et al. (85) developed and validated DeepSLE, a DL system that detects SLE and its complications from retinal fundus images alone, across diverse multi-ethnic populations. This non-invasive approach offers a cost-effective screening solution with particular relevance for primary care and low-resource settings. Despite these advances, the application of DL in SLE remains relatively limited compared with other complex immune-mediated diseases such as rheumatoid arthritis, ulcerative colitis, multiple sclerosis, and Hashimoto’s thyroiditis (86–92). However, existing studies collectively demonstrate substantial untapped potential. By integrating multi-omics, single-cell, imaging, and clinical data, DL approaches have reinforced the view that SLE is not a single disease but a collection of distinct molecular endotypes driven by different immune programs, including interferon (IFN)-, and lymphocyte-centered pathways. These insights link molecular endotypes to disease activity, organ involvement, flare risk, and treatment response, providing a foundation for precision diagnosis, prognosis, and targeted therapy in lupus.

## AI applications across molecular and immunologic phenotyping modalities in SLE

To contextualize AI applications in SLE, it is important to distinguish between conceptual potential and the current maturity of each molecular phenotyping domain. While AI has been widely proposed for multi-omics integration, progress remains uneven across data types, with transcriptomics representing the most advanced modality and other layers at varying stages of development. A modality-based summary of AI applications across genomics, epigenomics, chromatin accessibility, transcriptomics, proteomics, and multi-omics integration, highlighting key applications, findings and limitations is provided in **Table 2** along with representative references.

### Challenges and limitations of AI

Realizing the clinical potential of AI-driven multi-omics in SLE requires addressing several interconnected challenges. Data quality and study design are foundational: reliable models depend on well-curated, standardized datasets with harmonized protocols for sample collection, processing, and metadata annotation, ideally drawn from the same patient and time point. Without this rigor, AI models risk amplifying technical noise and producing non-reproducible results (93). Large-scale patient datasets also raise ethical and governance concerns, including data privacy, informed consent, and equitable representation. European League Against Rheumatism (EULAR) guidelines emphasize transparency, data quality, interoperability, and bias avoidance as core principles for responsible AI use in rheumatic diseases, ensuring that models are both technically robust and generalizable across diverse populations (94). Validation is equally important: molecular signatures identified through AI must be replicated across independent cohorts, platforms, and populations before they can inform clinical practice. Reproducibility remains a central challenge, requiring external validation, prospective study designs, and functional confirmation of candidate biomarkers (95). Finally, while multi-omics analyses have uncovered numerous molecular signatures in SLE, few have been translated into validated clinical assays. Oncology and chronic disease research offer instructive examples, where integrative DL models have enabled tumor classification, prognostication, and risk stratification (96, 97). These efforts provide a roadmap for SLE, where combining multi-omics with clinically accessible data and prospective validation will be essential to bridge the gap between discovery and implementation.

### Foundation models and future directions

FMs mark a radical shift in precision medicine. By pretraining on large immune atlases and multimodal biomedical resources, these models capture conserved immune cell states and activation programs that recur across tissues, diseases, and populations, enabling biological insight and generalization in SLE. The power of these models comes from learning transferable biological representations from large, heterogeneous datasets, rather than being trained for narrowly defined tasks within single cohorts.

When applied to autoimmune disease, this paradigm highlights a key insight that was difficult to capture using traditional ML models: the molecular heterogeneity of SLE reflects distinct immune subtypes defined by recurring combinations of shared immune programs, such as IFN signaling, B cell activity, and myeloid activation, rather than by broad, lupus-specific immune pathways. In contrast to SLE-only models that often capture cohort- or ancestry-dependent signals, foundation-style pretraining enables more consistent identification of biologically meaningful immune states across different datasets and patient populations. Looking ahead, the greatest impact of FMs in SLE is likely to come from integrating molecular endotyping, disease heterogeneity, and therapeutic stratification within a single, transferable framework grounded in broadly conserved immune biology.

Evidence from other disease domains demonstrates how FMs can be applied in practice and achieve strong performance. In computational pathology, vision FMs such as *Virchow* have achieved high accuracy in pan-cancer detection, with specimen-level area under the curve (AUC) of ~0.95 and robust generalization across datasets (45). In cardiovascular medicine, models such as *EchoCLIP*, trained on over one million echocardiography studies, have shown strong performance across multiple diagnostic tasks without task-specific retraining (46). In transcriptomics, models such as *Geneformer* and *Bulkformer* have demonstrated the ability to learn biologically meaningful gene-expression representations, enabling disease gene prioritization, pathway inference, and cross-cohort prediction, with *Bulkformer* additionally trained on large-scale bulk RNA-sequencing datasets that include lupus cohorts (12, 13). Similarly, single-cell foundation models such as scGPT and CellFM support cell-type annotation, multi-omic integration, and transfer learning across datasets (14, 47).

An emerging immunology-related FM, *ImmuneFM*, is pre-trained on large-scale single-cell cytometry data from over 100 million cells across 53 immunology studies. It learns transferable immune-cell representations that can be fine-tuned for tasks such as autoimmune disease classification and immune profiling, outperforming conventional methods. The model is already trained with immunophenotyping CyTOF data from lupus patients, which can further be utilized in unique marker discovery, patient stratification, and personalized therapy (48).

These examples highlight a key principle relevant to SLE: FMs can learn shared biological representations and be adapted to multiple clinical tasks, including diagnosis, patient stratification, disease activity prediction, and therapeutic response. In lupus, such models could integrate multi-omics and clinical data to identify conserved immune states and enable unified, task-agnostic prediction frameworks. This approach addresses a major limitation of current studies, where separate models are developed using small cohorts, thereby improving robustness and generalizability. However, FMs in SLE remain at an early stage, with limitations in zero-shot performance and susceptibility to batch effects. Moreover, most AI models lack prospective validation, and clinical translation will require standardized study design, high-quality multi-omics datasets, and rigorous validation across independent cohorts.

## Conclusion

AI has deepened our understanding of SLE from a single, uniform disease into a spectrum of biologically distinct immune endotypes. By integrating multimodal molecular and clinical data, AI-driven analyses have consistently shown that patients with similar clinical presentations can differ fundamentally in their underlying immune signatures such as IFN-, B-cell–, or myeloid-dominant states, providing a mechanistic explanation for the marked heterogeneity in disease course and treatment response (**Figure 1**). These findings establish molecular stratification as a biological reality rather than an analytical artifact and create a rational foundation for precision therapy in lupus.

At the same time, translation into routine clinical practice remains limited. Most AI-derived biomarkers and prediction models are retrospective, cohort-specific, and insufficiently validated, with performance often affected by technical variability, ancestry composition, and lack of standardized workflows. Bridging this gap will require coordinated action: prospective, multicentered validation of existing tools; harmonization of molecular assays and biomarker definitions; and intentional inclusion of diverse populations and upfront handling of confounding factors.

Looking forward, FMs offer a promising alternative to overcome these limitations by learning transferable immune representations from large, diverse datasets and enabling consistent molecular stratification across cohorts and clinical applications. To realize the potential of these models, the scientific and clinical communities must align on study design, clinical trials, and data generation with this paradigm, collecting longitudinal, multimodal data and embedding molecular endotyping into therapeutic decision-making. Ultimately, the value of AI in SLE lies not in incremental gains in prediction accuracy, but in enabling a transition from empiric, trial-and-error treatment to mechanistically informed, patient-stratified care, aiming for maximum efficacy with minimal toxicity, where therapy is guided by an individual’s immune biology rather than by clinical phenotype alone. AI has substantially advanced molecular and immunologic phenotyping in SLE, with transcriptomics and proteomics leading the field in terms of maturity and performance. However, significant gaps remain in integrating multi-omics data, validating models across diverse populations, and translating findings into clinical decision-making tools. Addressing these challenges will be essential for realizing the full potential of AI-driven precision medicine in SLE.

## Literature search strategy

A systematic literature search was conducted across PubMed/MEDLINE, Scopus, Web of Science, and bioRxiv/medRxiv, covering publications across all time periods. Priority was given to recent English-language articles (last 5 years) in peer-reviewed journals for evolving AI topics, while older literature was referenced for well-established biological concepts.

Search queries combined MeSH terms and free-text keywords, including but not limited to: “systemic lupus erythematosus” OR “lupus nephritis” OR “SLE” AND “foundation model” OR “large language model” OR “transformer” OR “deep learning” OR “machine learning” OR “artificial intelligence” AND “disease stratification” OR “precision medicine” OR “personalized medicine” OR “biomarker” OR “multiomics”, with Boolean operators applied to maximize retrieval sensitivity.

Titles and abstracts were screened against predefined search criteria, prioritizing studies that reported AI or FM applications in SLE-related disease stratification, outcome prediction, or treatment personalization. Forward and backward citation tracking was performed to minimize retrieval gaps. To limit selection bias, search queries were not framed around anticipated outcomes or specific algorithmic frameworks.

## References

[1] Kruta J Carapito R Trendelenburg M Martin T Rizzi M Voll RE . Machine learning for precision diagnostics of autoimmunity. Sci Rep. (2024) 14:27848. doi: 10.1038/S41598-024-76093-7. PMID: 39537649 PMC11561187

[2] Yaung KN Yeo JG Kumar P Wasser M Chew M Ravelli A . Artificial intelligence and high-dimensional technologies in the theragnosis of systemic lupus erythematosus. Lancet Rheumatol. (2023) 5:e151–e165. doi: 10.1016/S2665-9913(23)00010-3. PMID: 38251610

[3] Asnicar F Thomas AM Passerini A Waldron L Segata N . Machine learning for microbiologists. Nat Rev Microbiol. (2024) 22:191–205. doi: 10.1038/S41579-023-00984-1. PMID: 37968359 PMC11980903

[4] Zhang J Ma L Deng H Yi W Tohtihan A Tang X . Multi-omics integration identifies NK cell-mediated cytotoxicity as a therapeutic target in systemic lupus erythematosus. Front Immunol. (2025) 16:1580540. doi: 10.3389/FIMMU.2025.1580540. PMID: 40433370 PMC12106370

[5] Garcia-Bañol DF Arias-Choles AM Aldana-Peréz S Aroca-Martínez GJ Musso CG Navarro-Quiroz R . Machine learning in lupus nephritis: bridging prediction models and clinical decision-making towards personalized nephrology. Front Med (Lausanne). (2025) 12:1686057. doi: 10.3389/FMED.2025.1686057. PMID: 41234906 PMC12605162

[6] Adamichou C Genitsaridi I Nikolopoulos D Nikoloudaki M Repa A Bortoluzzi A . Lupus or not? SLE Risk Probability Index (SLERPI): a simple, clinician-friendly machine learning-based model to assist the diagnosis of systemic lupus erythematosus. Ann Rheum Dis. (2021) 80:758–66. doi: 10.1136/annrheumdis-2020-219069. PMID: 33568388 PMC8142436

[7] Figgett WA Monaghan K Ng M Alhamdoosh M Maraskovsky E Wilson NJ . Machine learning applied to whole-blood RNA-sequencing data uncovers distinct subsets of patients with systemic lupus erythematosus. Clin Transl Immunol. (2019) 8:e01093. doi: 10.1002/CTI2.1093. PMID: 31921420 PMC6946916

[8] Li X Huo Y Wang Z . Screening of potential biomarkers of system lupus erythematosus based on WGCNA and machine learning algorithms. Med (United States). (2023) 102:E36243. doi: 10.1097/MD.0000000000036243. PMID: 38013304 PMC10681579

[9] Chen W Wang X Huang G Sheng Q Zhou E . Identification of cellular senescence-related genes as biomarkers for lupus nephritis based on bioinformatics. Front Genet. (2025) 16:1551450. doi: 10.3389/fgene.2025.1551450. PMID: 40290492 PMC12021929

[10] Huang S Chen Y Song Y Wu K Chen T Zhang Y . Deep learning model to predict lupus nephritis renal flare based on dynamic multivariable time-series data. BMJ Open. (2024) 14:e071821. doi: 10.1136/BMJOPEN-2023-071821. PMID: 38485471 PMC10941130

[11] Stojanowski J Konieczny A Rydzyńska K Kasenberg I Mikołajczak A Gołębiowski T . Artificial neural network - an effective tool for predicting the lupus nephritis outcome. BMC Nephrol. (2022) 23:381. doi: 10.1186/S12882-022-02978-2. PMID: 36443678 PMC9706924

[12] Theodoris CV Xiao L Chopra A Chaffin MD Al Sayed ZR Hill MC . Transfer learning enables predictions in network biology. Nature. (2023) 618:616–24. doi: 10.1038/s41586-023-06139-9. PMID: 37258680 PMC10949956

[13] Kang B RF 1 Yi M Cui C Cui Q . A large-scale foundation model for bulk transcriptomes. bioRxiv. (2025), 2025.06.11.659222. doi: 10.1101/2025.06.11.659222. PMID: 38621210

[14] Cui H Wang C Maan H Pang K Luo F Duan N . scGPT: toward building a foundation model for single-cell multi-omics using generative AI. Nat Methods. (2024) 21:1470–80. doi: 10.1038/s41592-024-02201-0. PMID: 38409223

[15] Kalfon J Samaran J Peyré G Cantini L . scPRINT: pre-training on 50 million cells allows robust gene network predictions. Nat Commun. (2025) 16:3607. doi: 10.1038/s41467-025-58699-1. PMID: 40240364 PMC12003772

[16] Kegerreis B Catalina MD Bachali P Geraci NS Labonte AC Zeng C . Machine learning approaches to predict lupus disease activity from gene expression data. Sci Rep. (2019) 9:9617. doi: 10.1038/S41598-019-45989-0. PMID: 31270349 PMC6610624

[17] Guy RT Santago P Langefeld CD . Bootstrap aggregating of alternating decision trees to detect sets of SNPs that associate with disease. Genet Epidemiol. (2012) 36:99–106. doi: 10.1002/GEPI.21608. PMID: 22851473 PMC3769952

[18] Ma W Lau YL Yang W Wang YF . Random forests algorithm boosts genetic risk prediction of systemic lupus erythematosus. Front Genet. (2022) 13:902793. doi: 10.3389/fgene.2022.902793. PMID: 36046232 PMC9421562

[19] Chung CW Chou SC Hsiao TH Zhang GJ Chung YF Chen YM . Machine learning approaches to identify systemic lupus erythematosus in anti-nuclear antibody-positive patients using genomic data and electronic health records. Biodata Min. (2024) 17:1. doi: 10.1186/s13040-023-00352-y. PMID: 38183082 PMC10770905

[20] Bosello SL Ortolan A Lanzo L Lilli L Antenucci L Cerasuolo PG . Identification of clinical phenotypes and disease trajectories in SLE using AI through a natural language processing framework. Rheumatol (Oxford). (2026) 65:keag035. doi: 10.1093/rheumatology/keag035. PMID: 41701167

[21] Wang J Dang X Wu X Xiang Z Li Y Fu Y . DNA methylation of IFI44L as a potential blood biomarker for childhood-onset systemic lupus erythematosus. Pediatr Res. (2024) 96:494–501. doi: 10.1038/s41390-024-03135-1. PMID: 38514858 PMC11343705

[22] Zhou X Zhou S Li Y . An updated review on abnormal epigenetic modifications in the pathogenesis of systemic lupus erythematosus. Front Immunol. (2025) 15:1501783. doi: 10.3389/fimmu.2024.1501783. PMID: 39835138 PMC11743643

[23] Castellini-Pérez O Povedano E Barturen G Martínez-Bueno M Iakovliev A Kerick M . Molecular subtypes explain lupus epigenomic heterogeneity unveiling new regulatory genetic risk variants. NPJ Genom Med. (2024) 9:38. doi: 10.1038/s41525-024-00420-0. PMID: 39013887 PMC11252280

[24] Horton MK Nititham J Taylor KE Katz P Ye CJ Yazdany J . Changes in DNA methylation are associated with systemic lupus erythematosus flare remission and clinical subtypes. Clin Epigenet. (2024) 16:181. doi: 10.1186/s13148-024-01792-x. PMID: 39696438 PMC11656870

[25] Guo C Liu Q Zong D Zhang W Zuo Z Yu Q . Single-cell transcriptome profiling and chromatin accessibility reveal an exhausted regulatory CD4+ T cell subset in systemic lupus erythematosus. Cell Rep. (2022) 41. doi: 10.1016/j.celrep.2022.111606. PMID: 36351407

[26] Beigel K Wang XM Song L Maurer K Breen C Taylor D . Comparison of cell type and disease subset chromatin modifications in SLE. Clin Epigenet. (2024) 16:159. doi: 10.1186/s13148-024-01754-3. PMID: 39543716 PMC11566291

[27] Leventhal EL Daamen AR Grammer AC Lipsky PE . An interpretable machine learning pipeline based on transcriptomics predicts phenotypes of lupus patients. iScience. (2023) 26. doi: 10.1016/j.isci.2023.108042. PMID: 37860757 PMC10582499

[28] Yones SA Annett A Stoll P Diamanti K Holmfeldt L Barrenäs CF . Interpretable machine learning identifies paediatric Systemic Lupus Erythematosus subtypes based on gene expression data. Sci Rep. (2022) 12:7433. doi: 10.1038/S41598-022-10853-1. PMID: 35523803 PMC9076598

[29] Toro-Domínguez D Lopez-Domínguez R García Moreno A Villatoro-García JA Martorell-Marugán J Goldman D . Differential treatments based on drug-induced gene expression signatures and longitudinal systemic lupus erythematosus stratification. Sci Rep. (2019) 9:15502. doi: 10.1038/s41598-019-51616-9. PMID: 31664045 PMC6820741

[30] Hubbard EL Bachali P Kingsmore KM He Y Catalina MD Grammer AC . Analysis of transcriptomic features reveals molecular endotypes of SLE with clinical implications. Genome Med. (2023) 15:84. doi: 10.1186/S13073-023-01237-9. PMID: 37845772 PMC10578040

[31] Qiao J Zhang SX Chang MJ Zhao R Song S Hao JW . Deep stratification by transcriptome molecular characters for precision treatment of patients with systemic lupus erythematosus. Rheumatol (Oxford). (2023) 62:2574–84. doi: 10.1093/rheumatology/keac625. PMID: 36308437

[32] He J Liu Z Tang X . A deep learning model for predicting systemic lupus erythematosus-associated epitopes. BMC Med Inform Decis Mak. (2025) 25:230. doi: 10.1186/s12911-025-03056-x. PMID: 40598008 PMC12220259

[33] Choi MY Chen I Clarke AE Fritzler MJ Buhler KA Urowitz M . Machine learning identifies clusters of longitudinal autoantibody profiles predictive of systemic lupus erythematosus disease outcomes. Ann Rheum Dis. (2023) 82:927–36. doi: 10.1136/ard-2022-223808. PMID: 37085289 PMC11293954

[34] Guthridge JM Lu R Tran LTH Arriens C Aberle T Kamp S . Adults with systemic lupus exhibit distinct molecular phenotypes in a cross-sectional study. EClinicalMedicine. (2020) 20. doi: 10.1016/j.eclinm.2020.100291. PMID: 32154507 PMC7058913

[35] Huang Z Shi Y Cai B Wang L Wu Y Ying B . MALDI-TOF MS combined with magnetic beads for detecting serum protein biomarkers and establishment of boosting decision tree model for diagnosis of systemic lupus erythematosus. Rheumatol (Oxford). (2009) 48:626–31. doi: 10.1093/RHEUMATOLOGY/KEP058. PMID: 19389822

[36] Pattanaik SS Das BK Tripathy R Prusty BK Parida MK Tripathy SR . Machine learning identifies cytokine signatures of disease severity and autoantibody profiles in systemic lupus erythematosus - a pilot study. Sci Rep. (2024) 14:28765. doi: 10.1038/s41598-024-79978-9. PMID: 39567666 PMC11579361

[37] Su KYC Reynolds JA Reed R Da Silva R Kelsall J Baricevic-Jones I . Proteomic analysis identifies subgroups of patients with active systemic lupus erythematosus. Clin Proteomics. (2023) 20:29. doi: 10.1186/s12014-023-09420-1. PMID: 37516862 PMC10385905

[38] Izuka S Komai T Itamiya T Ota M Nagafuchi Y Shoda H . Machine learning-driven immunophenotypic stratification of mixed connective tissue disease, corroborating the clinical heterogeneity. Rheumatol (Oxford). (2025) 64:1409–16. doi: 10.1093/rheumatology/keae158. PMID: 38479808 PMC11879315

[39] Curion F Theis FJ . Machine learning integrative approaches to advance computational immunology. Genome Med. (2024) 16:80. doi: 10.1186/s13073-024-01350-3. PMID: 38862979 PMC11165829

[40] Machine Learning–Defined Subtypes Of Systemic Lupus Erythematosus Identify Distinct Immunologic And Molecular Signatures - Acr Meeting Abstracts. Available online at: https://acrabstracts.org/abstract/machine-learning-defined-subtypes-of-systemic-lupus-erythematosus-identify-distinct-immunologic-and-molecular-signatures/. (Accessed October 2025).

[41] Trzupek D Lee M Hamey F Wicker LS Todd JA Ferreira RC . Single-cell multi-omics analysis reveals IFN-driven alterations in T lymphocytes and natural killer cells in systemic lupus erythematosus. Wellcome Open Res. (2022) 6:149. doi: 10.12688/WELLCOMEOPENRES.16883.2. PMID: 35509371 PMC9046903

[42] Li Y Ma C Liao S Qi S Meng S Cai W . Combined proteomics and single cell RNA-sequencing analysis to identify biomarkers of disease diagnosis and disease exacerbation for systemic lupus erythematosus. Front Immunol. (2022) 13:969509. doi: 10.3389/fimmu.2022.969509. PMID: 36524113 PMC9746895

[43] Zhou H Li X Zhang Y Wei F Liu Z Zhao Y . Machine learning combined multi-omics analysis to explore key oxidative stress features in systemic lupus erythematosus. Front Immunol. (2025) 16:1567466. doi: 10.3389/fimmu.2025.1567466. PMID: 40625749 PMC12231464

[44] Fareed MM Shityakov S . Integrative machine learning of gene expression and DNA methylation for the accurate diagnosis of systemic lupus erythematosus and Sjögren’s syndrome. In Silico Res Biomedicine. (2025) 1:100123. doi: 10.1016/j.insi.2025.100123. PMID: 38826717

[45] Vorontsov E Bozkurt A Casson A Shaikovski G Zelechowski M Severson K . A foundation model for clinical-grade computational pathology and rare cancers detection. Nat Med. (2024) 30:2924–35. doi: 10.1038/s41591-024-03141-0. PMID: 39039250 PMC11485232

[46] Christensen M Vukadinovic M Yuan N Ouyang D . Vision-language foundation model for echocardiogram interpretation. Nat Med. (2024) 30:1481–8. doi: 10.1038/s41591-024-02959-y. PMID: 38689062 PMC11108770

[47] Zeng Y Xie J Shangguan N Wei Z Li W Su Y . CellFM: a large-scale foundation model pre-trained on transcriptomics of 100 million human cells. Nat Commun. (2025) 16:4679. doi: 10.1038/s41467-025-59926-5. PMID: 40393991 PMC12092794

[48] Ding S Bhattacharya S Butte AJ . ImmuneFM: Pre-training foundation model from cytometry data for immunology research. bioRxiv. (2025), 2025.07.09.664020. doi: 10.1101/2025.07.09.664020. PMID: 38621210

[49] Nikolopoulos D Loukogiannaki C Sentis G Garantziotis P Manolakou T Kapsala N . Disentangling the riddle of systemic lupus erythematosus with antiphospholipid syndrome: blood transcriptome analysis reveals a less-pronounced IFN-signature and distinct molecular profiles in venous versus arterial events. Ann Rheum Dis. (2024) 83:1132–43. doi: 10.1136/ard-2024-225664. PMID: 38609158 PMC11420729

[50] Chen Y Huang S Chen T Liang D Yang J Zeng C . Machine learning for prediction and risk stratification of lupus nephritis renal flare. Am J Nephrol. (2021) 52:152–60. doi: 10.1159/000513566. PMID: 33744876

[51] Accapezzato D Caccavale R Paroli MP Gioia C Nguyen BL Spadea L . Advances in the pathogenesis and treatment of systemic lupus erythematosus. Int J Mol Sci. (2023) 24:6578. doi: 10.3390/IJMS24076578. PMID: 37047548 PMC10095030

[52] Ghodke-Puranik Y Imgruet M Dorschner JM Shrestha P McCoy K Kelly JA . Novel genetic associations with interferon in systemic lupus erythematosus identified by replication and fine-mapping of trait-stratified genome-wide screen. Cytokine. (2020) 132:154631. doi: 10.1016/j.cyto.2018.12.014. PMID: 30685201 PMC7723062

[53] Lanata CM Paranjpe I Nititham J Taylor KE GianFrancesco M Paranjpe M . A phenotypic and genomics approach in a multi-ethnic cohort to subtype systemic lupus erythematosus. Nat Commun. (2019) 10:3902. doi: 10.1038/S41467-019-11845-Y. PMID: 31467281 PMC6715644

[54] Lazar S Kahlenberg JM . Systemic lupus erythematosus: new diagnostic and therapeutic approaches. Annu Rev Med. (2023) 74:339–52. doi: 10.1146/ANNUREV-MED-043021-032611. PMID: 35804480

[55] Usategui I Arroyo Y Torres AM Barbado J Mateo J . Systemic lupus erythematosus: how machine learning can help distinguish between infections and flares. Bioengineering (Basel). (2024) 11. doi: 10.3390/BIOENGINEERING11010090. PMID: 38247967 PMC11154352

[56] Zhan K Buhler KA Chen IY Fritzler MJ Choi MY . Systemic lupus in the era of machine learning medicine. Lupus Sci Med. (2024) 11. doi: 10.1136/LUPUS-2023-001140. PMID: 38443092 PMC11146397

[57] Allen ME Rus V Szeto GL . Leveraging heterogeneity in systemic lupus erythematosus for new therapies. Trends Mol Med. (2021) 27:152–71. doi: 10.1016/j.molmed.2020.09.009. PMID: 33046407 PMC8667782

[58] Abid N Manaye S Naushad H Cheran K Murthy C Bornemann EA . The safety and efficacy of rituximab and belimumab in systemic lupus erythematosus: A systematic review. Cureus. (2023) 15. doi: 10.7759/CUREUS.40719. PMID: 37485087 PMC10360028

[59] He J Li Z . An era of biological treatment in systemic lupus erythematosus. Clin Rheumatol. (2018) 37:1–3. doi: 10.1007/S10067-017-3933-X. PMID: 29234909 PMC5754454

[60] Steiger S Ehreiser L Anders J Anders HJ . Biological drugs for systemic lupus erythematosus or active lupus nephritis and rates of infectious complications. Evidence from large clinical trials. Front Immunol. (2022) 13:999704/PDF. doi: 10.3389/fimmu.2022.999704 36211360 PMC9538665

[61] Sinicato NA Postal M Appenzeller S Niewold TB . Defining biological subsets in systemic lupus erythematosus: progress toward personalized therapy. Pharmaceut Med. (2017) 31:81–8. doi: 10.1007/S40290-017-0178-6. PMID: 28827978 PMC5562038

[62] Garantziotis P Nikolakis D Doumas S Frangou E Sentis G Filia A . Molecular taxonomy of systemic lupus erythematosus through data-driven patient stratification: molecular endotypes and cluster-tailored drugs. Front Immunol. (2022) 13:860726/PDF. doi: 10.3389/fimmu.2022.860726 35615355 PMC9125979

[63] Demkova K Morris DL Vyse TJ . Genetics of SLE: does this explain susceptibility and severity across racial groups? Rheumatol (Oxford). (2023) 62:I15–I21. doi: 10.1093/RHEUMATOLOGY/KEAC695. PMID: 36583554 PMC10050932

[64] Kamen DL . Environmental influences on systemic lupus erythematosus expression. Rheumatic Dis Clinics North America. (2014) 40:401–12. doi: 10.1016/j.rdc.2014.05.003. PMID: 25034153 PMC4198387

[65] Toro-Domínguez D Martorell-Marugán J Martinez-Bueno M López-Domínguez R Carnero-Montoro E Barturen G . Scoring personalized molecular portraits identify Systemic Lupus Erythematosus subtypes and predict individualized drug responses, symptomatology and disease progression. Brief Bioinform. (2022) 23:bbac332. doi: 10.1093/BIB/BBAC332. PMID: 35947992 PMC9487588

[66] Bennett L Palucka AK Arce E Cantrell V Borvak J Banchereau J . Interferon and granulopoiesis signatures in systemic lupus erythematosus blood. J Exp Med. (2003) 197:711–23. doi: 10.1084/JEM.20021553. PMID: 12642603 PMC2193846

[67] Robl R Eudy A Bachali PS Rogers JL Clowse M Pisetsky D . Molecular endotypes of type 1 and type 2 SLE. Lupus Sci Med. (2023) 10. doi: 10.1136/LUPUS-2022-000861. PMID: 36720488 PMC9950972

[68] Moysidou GS Garantziotis P Sentis G Nikoleri D Malissovas N Nikoloudaki M . Molecular basis for the disease-modifying effects of belimumab in systemic lupus erythematosus and molecular predictors of early response: blood transcriptome analysis implicates the innate immunity and DNA damage response pathways. Ann Rheum Dis. (2025) 84:262–73. doi: 10.1136/ard-2024-226051. PMID: 39919899

[69] Siddiqi KZ Wilhelm TR Ulff-Møller CJ Jacobsen S . Cluster of highly expressed interferon-stimulated genes associate more with African ancestry than disease activity in patients with systemic lupus erythematosus. A systematic review of cross-sectional studies. Trans Res. (2021) 238:63–75. doi: 10.1016/j.trsl.2021.07.006. PMID: 34343626

[70] Van Vollenhoven RF Petri MA Cervera R Roth DA Ji BN Kleoudis CS . Belimumab in the treatment of systemic lupus erythematosus: high disease activity predictors of response. Ann Rheum Dis. (2012) 71:1343–9. doi: 10.1136/ANNRHEUMDIS-2011-200937. PMID: 22337213 PMC3396451

[71] Dimelow R Gillespie WR van Maurik A . Population model-based analysis of the memory B-cell response following belimumab therapy in the treatment of systemic lupus erythematosus. CPT Pharmacometrics Syst Pharmacol. (2023) 12:462–73. doi: 10.1002/PSP4.12919. PMID: 36852495 PMC10088083

[72] Simeoni M Yang S Tompson DJ Dimelow R . Longitudinal modeling of efficacy response in patients with lupus nephritis receiving belimumab. J Pharmacokinet Pharmacodyn. (2024) 51:289–301. doi: 10.1007/S10928-024-09907-W. PMID: 38551711 PMC11136851

[73] Li H Ju B Luo J Zhu L Zhang J Hu N . Type I interferon-stimulated genes predict clinical response to belimumab in systemic lupus erythematosus. Eur J Pharmacol. (2025) 987:177204. doi: 10.1016/j.ejphar.2024.177204. PMID: 39672224

[74] Wilkinson C Henderson RB Jones-Leone AR Flint SM Lennon M Levy RA . The role of baseline BLyS levels and type 1 interferon-inducible gene signature status in determining belimumab response in systemic lupus erythematosus: a post hoc meta-analysis. Arthritis Res Ther. (2020) 22:102. doi: 10.1186/S13075-020-02177-0. PMID: 32366280 PMC7197114

[75] Morand EF Furie R Tanaka Y Bruce IN Askanase AD Richez C . Trial of anifrolumab in active systemic lupus erythematosus. N Engl J Med. (2020) 382:211–21. doi: 10.1056/NEJMOA1912196. PMID: 31851795

[76] Saegusa K Tsuchida Y Komai T Tsuchiya H Fujio K . Advances in targeted therapy for systemic lupus erythematosus: current treatments and novel approaches. Int J Mol Sci. (2025) 26:929. doi: 10.3390/IJMS26030929. PMID: 39940698 PMC11816971

[77] Murray SG Avati A Schmajuk G Yazdany J . Automated and flexible identification of complex disease: building a model for systemic lupus erythematosus using noisy labeling. J Am Med Inform Assoc. (2019) 26:61–5. doi: 10.1093/JAMIA/OCY154. PMID: 30476175 PMC6308013

[78] Frangou E Garantziotis P Grigoriou M Banos A Nikolopoulos D Pieta A . Cross-species transcriptome analysis for early detection and specific therapeutic targeting of human lupus nephritis. Ann Rheum Dis. (2022) 81:1409–19. doi: 10.1136/annrheumdis-2021-222069. PMID: 35906002 PMC9484391

[79] Jorge A Castro VM Barnado A Gainer V Hong C Cai T . Identifying lupus patients in electronic health records: Development and validation of machine learning algorithms and application of rule-based algorithms. Semin Arthritis Rheum. (2019) 49:84–90. doi: 10.1016/j.semarthrit.2019.01.002. PMID: 30665626 PMC6609504

[80] Turner CA Jacobs AD Marques CK Oates JC Kamen DL Anderson PE . Word2Vec inversion and traditional text classifiers for phenotyping lupus. BMC Med Inform Decis Mak. (2017) 17:126. doi: 10.1186/S12911-017-0518-1. PMID: 28830409 PMC5568290

[81] Jorge AM Smith D Wu Z Chowdhury T Costenbader K Zhang Y . Exploration of machine learning methods to predict systemic lupus erythematosus hospitalizations. Lupus. (2022) 31:1296–305. doi: 10.1177/09612033221114805. PMID: 35835534 PMC9547899

[82] Ayoub I Wolf BJ Geng L Song H Khatiwada A Tsao BP . Prediction models of treatment response in lupus nephritis. Kidney Int. (2022) 101:379–89. doi: 10.1016/j.kint.2021.11.014. PMID: 34871620 PMC8792241

[83] Hao Y Cheng C Li J Li H Di X Zeng X . Multimodal integration in health care: Development with applications in disease management. J Med Internet Res. (2025) 27:e76557. doi: 10.2196/76557. PMID: 40840463 PMC12370271

[84] Wang G Zhao J Lin Y Liu T Zhao Y Zhao H . scMODAL: a general deep learning framework for comprehensive single-cell multi-omics data alignment with feature links. Nat Commun. (2025) 16:4994. doi: 10.1038/S41467-025-60333-Z. PMID: 40442129 PMC12122792

[85] Li T Lin S Guan Z Zhou Y Zeng D Wang Z . A deep learning system for detecting systemic lupus erythematosus from retinal images. Cell Rep Med. (2025) 6. doi: 10.1016/j.xcrm.2025.102203. PMID: 40570853 PMC12281353

[86] He X Wang M Zhao C Wang Q Zhang R Liu J . Deep learning-based automatic scoring models for the disease activity of rheumatoid arthritis based on multimodal ultrasound images. Rheumatol (Oxford). (2024) 63:866–73. doi: 10.1093/RHEUMATOLOGY/KEAD366. PMID: 37471602

[87] Venalainen MS Biehl A Holstila M Kuusalo L Elo LL . Deep learning enables automatic detection of joint damage progression in rheumatoid arthritis-model development and external validation. Rheumatol (Oxford). (2025) 64:1068–76. doi: 10.1093/RHEUMATOLOGY/KEAE215. PMID: 38597875 PMC11879318

[88] Kim JE Choi YH Lee YC Seong G Song JH Kim TJ . Deep learning model for distinguishing Mayo endoscopic subscore 0 and 1 in patients with ulcerative colitis. Sci Rep. (2023) 13:11351. doi: 10.1038/S41598-023-38206-6. PMID: 37443370 PMC10344868

[89] Qi J Ruan G Ping Y Xiao Z Liu K Cheng Y . Development and validation of a deep learning-based approach to predict the Mayo endoscopic score of ulcerative colitis. Therap Adv Gastroenterol. (2023) 16:17562848231170945. doi: 10.1177/17562848231170945. PMID: 37251086 PMC10214058

[90] Wiltgen T McGinnis J Schlaeger S Kofler F Voon CC Berthele A . LST-AI: A deep learning ensemble for accurate MS lesion segmentation. NeuroImage Clin. (2024) 42:103611. doi: 10.1016/j.nicl.2024.103611. PMID: 38703470 PMC11088188

[91] Dereskewicz E La Rosa F dos Santos Silva J Sizer E Kohli A Wynen M . FLAMeS: A robust deep learning model for automated multiple sclerosis lesion segmentation. medRxiv. (2025). doi: 10.1101/2025.05.19.25327707. PMID: 40937688 PMC12426979

[92] Zhang Q Zhang S Pan Y Sun L Li J Qiao Y . Deep learning to diagnose Hashimoto’s thyroiditis from sonographic images. Nat Commun. (2022) 13:3759. doi: 10.1038/S41467-022-31449-3. PMID: 35768466 PMC9243092

[93] Ouhmouk M Baichoo S Abik M . Challenges in AI-driven multi-omics data analysis for oncology: Addressing dimensionality, sparsity, transparency and ethical considerations. Inform Med Unlocked. (2025) 57:101679. doi: 10.1016/j.imu.2025.101679. PMID: 38826717

[94] Gossec L Kedra J Servy H Pandit A Stones S Berenbaum F . EULAR points to consider for the use of big data in rheumatic and musculoskeletal diseases. Ann Rheum Dis. (2020) 79:69–76. doi: 10.1136/annrheumdis-2019-215694. PMID: 31229952

[95] Perng W Aslibekyan S . Find the needle in the haystack, then find it again: Replication and validation in the ‘omics era. Metabolites. (2020) 10:1–13. doi: 10.3390/metabo10070286. PMID: 32664690 PMC7408356

[96] Sartori F Codicè F Caranzano I Rollo C Birolo G Fariselli P . A comprehensive review of deep learning applications with multi-omics data in cancer research. Genes (Basel). (2025) 16:648. doi: 10.3390/genes16060648. PMID: 40565540 PMC12191839

[97] Dong Z Li P Jiang Y Wang Z Fu S Che H . Integrative multi-omics and routine blood analysis using deep learning: Cost-effective early prediction of chronic disease risks. Adv Sci (Weinh). (2025) 12:2412775. doi: 10.1002/advs.202412775. PMID: 40171841 PMC12165040

